# Rifampin resistance and diabetes mellitus in a cross-sectional study of adult patients in rural South India

**DOI:** 10.1186/s12879-015-1204-5

**Published:** 2015-10-26

**Authors:** Saurabh Mehta, Elaine Ann Yu, Syed Fazil Ahamed, Wesley Bonam, John Kenneth

**Affiliations:** Division of Nutritional Sciences, Cornell University, 314 Savage Hall, Ithaca, NY 14853 USA; Arogyavaram Medical Centre, Andhra Pradesh, India; Division of Infectious Diseases, St. John’s Research Institute, Bangalore, Karnataka India

**Keywords:** Tuberculosis, Drug resistance, Rifampin, Diabetes, India, Diagnostics, South Asia

## Abstract

**Background:**

Despite increasing reports of the linkage between diabetes and tuberculosis (TB), there is limited information regarding diabetes and TB drug resistance.

**Methods:**

In this cross-sectional study, sputum and blood samples were collected from 304 adult patients in rural Andhra Pradesh. Rifampin resistance was assessed by Xpert MTB/RIF (Xpert), and diabetes status was based on self-report. Additionally, samples were assayed by acid-fast bacilli sputum smear microscopy (AFB) and QuantiFERON-TB Gold In-Tube (QFT-G), in order to compare relative diagnostic performances.

**Results:**

Among patients with confirmed TB (*n* = 194), diabetes was associated with 3.0-fold higher risk of rifampin resistance (95 % CI 1.3–6.7). Considering Xpert MTB/RIF the gold standard, AFB had lower sensitivity (72.2 vs. 82.5 %) and higher specificity (96.4 vs. 37.0 %) compared to QFT-G for diagnosing TB.

**Conclusions:**

The increased risk of rifampin resistance in patients with diabetes highlights the need for integrated diabetes surveillance in TB programs, particularly in settings undergoing the epidemiological transition.

**Electronic supplementary material:**

The online version of this article (doi:10.1186/s12879-015-1204-5) contains supplementary material, which is available to authorized users.

## Background

The synergistic interactions between diabetes and tuberculosis (TB) are widely recognized [1]. Studies among patients with TB report associations between diabetes and adverse outcomes, including mortality, TB treatment failure and relapse [2]. Moreover, diabetes is associated with a 3-fold increased risk of active TB, according to a systematic review [3].

Globally, TB drug resistance is a growing challenge to improving TB control; estimates show 480,000 incident cases of multidrug-resistant TB (MDR-TB) in 2013 [4]. In 2013, over 50 % of MDR-TB cases were reported in India, China, and the Russian Federation [4]. Worldwide, the treatment success rate of MDR-TB (48 %) is significantly lower than that among new TB cases (86 %) [4].

Preliminary evidence from pharmacokinetics studies suggests that suggests that the linkage between diabetes and TB drug resistance is biologically plausible. One study showed reduced rifampin exposure among patients with TB-diabetes coinfections, compared to those with TB [5]. Furthermore, another study found that low absorption of anti-TB treatment (according to area under the curve [AUC]) was associated with increased drug resistance [6].

Key research gaps include determining the clinical relevance of potential effects of diabetes on TB drug resistance, particularly in settings undergoing the epidemiological transition [7]. During the shift from infectious to chronic diseases, certain geographic areas may still have higher prevalence of TB and simultaneously rising diabetes incidence. Therefore, our primary objective was to assess the association between diabetes mellitus and rifampin resistance among patients with active TB in South India. Secondarily, we compared the performance of Xpert MTB/RIF (Xpert) with acid-fast bacilli sputum smear microscopy (AFB) and QuantiFERON-TB Gold In-Tube (QFT-G).

## Methods

### Study population

In this secondary analysis of cross-sectional data, 304 adult patients were serially enrolled from an outpatient clinic of a rural hospital in Andhra Pradesh during 2012–13. Hospital physicians referred patients with clinical signs and symptoms of TB. Sputum and blood samples were collected from all suspected cases of TB, per standard of care. Demographic information and clinical history (including vaccinations and comorbidities) were collected from patient interviews.

The study protocol received approval from the institutional review board at St. John’s Research Institute (Bangalore, Karnataka, India). All study participants provided informed consent prior to any data collection. All analyses occurred subsequent to data de-identification (Additional file 1).

### Assessment of outcomes

Active and latent TB were assessed in sputum and blood samples by three techniques: Xpert (Cepheid, Sunnyvale, California, United States), AFB, and QFT-G (Cellestis Limited, Carnegie, Victoria, Australia). The presence of *Mycobacterium tuberculosis* (*MTB*) in sputum was evaluated by Xpert and AFB. For Xpert assays, sputum specimens were decontaminated with N-acetyl-L-cysteine-sodium hydroxide, and processed according to manufacturer instructions. As a fully automated real-time multiplex polymerase chain reaction assay that is based on the GeneXpert platform, Xpert also assessed rifampin resistance (via *rpoB* gene mutations). AFB was based on standard protocol for Ziehl-Neelsen staining with conventional light microscopy.

Additionally, QFT-G measured the interferon-gamma response triggered by incubation with two *MTB* antigens (early secretory antigenic target-6; culture filtrate protein-10). QFT-G is recommended as an aid for diagnosing TB infection, as it can serve as an alternative to tuberculin skin tests but does not distinguish between latent versus active TB. For the QFT-G assay, blood was initially collected in QFT-G tubes. Samples were centrifuged to obtain plasma, processed according to manufacturer protocol, and assessed by enzyme-linked immunosorbent assay.

### Assessment of exposure and other covariates

Diabetes status was based on patient self-report. Human immunodeficiency virus (HIV) status was assessed by a rapid HIV test (TRI-DOT; J. Mitra & Co. Pvt. Ltd.; New Delhi, India), per standard Revised National Tuberculosis Control Programme guidelines for individuals with suspected TB.

### Statistical analyses

Relative diagnostic performance was assessed by calculating sensitivity, specificity, predictive values, and AUC (by receiver operator characteristic [ROC] curve visualization). Xpert results were considered as the gold standard for comparing conventional TB diagnostics. Study participants were categorized as either previous or new cases, based on whether anti-TB treatment was previously received. Statistical analyses were conducted with SAS software (version 9.4; SAS Institute Inc., Cary, NC, USA); two-tailed P-values <0.05 were considered statistically significant.

Patient demographic and health characteristics were reported as means (standard deviations [SDs]) or percentages. Group differences were assessed by t-tests (continuous variables with normal distribution; Satterwaite method for unequal variance) or Mantel-Haenszel χ^2^-tests (categorical variables). Potential covariates were identified *a priori*, based on previous literature. Initially, associations between potential covariates were assessed by univariate log-binomial regression models; variables with *p* < 0.2 were included in the full model. Subsequently, only variables with *p* < 0.05 were retained in the final multivariate log-binomial regression model.

## Results

Among 304 study participants with suspected TB, 63.8 % had TB confirmed by Xpert, 10.9 % had diabetes, and 2.6 % were living with HIV (Table 1). Overall, 23.7 % were female, which did not differ significantly when stratified by diabetes (*p* = 0.72) or TB (*p* = 0.41). The average age of patients was 38.3 years (SD 11.5). Patients with diabetes were older than those without diabetes (*p* = 0.01); those with and without TB (based on Xpert) were similar in age (*p* = 0.08). The majority (87.5 %) had never previously received anti-TB treatment.

**Table 1:** Demographic and baseline health characteristics of patients^a^

	Overall		TB^b^			
			Y		N	
			*n = 194*		*n = 110*	
	N	Mean (SD)	Mean	SD	Mean	SD
Age^c^	304	38.3 (11.5)	37.4	11.3	39.8	11.6
	N	%	N	%	N	%
Female	72	23.7	43	22.2	29	26.4
Male	232	76.3	151	77.8	81	73.6
Time Since Previous TB Treatment (years)						
1	11	3.6	9	4.6	2	1.8
1.5	1	0.3	1	0.5	0	0.0
2	10	3.3	7	3.6	3	2.7
3	8	2.6	4	2.1	4	3.6
4	3	1.0	1	0.5	2	1.8
5	4	1.3	2	1.0	2	1.8
7	1	0.3	0	0.0	1	0.9
None	266	87.5	170	87.6	96	87.3
Rifampin Resistance						
Y	---	---	21	10.8	---	---
N	---	---	172	88.7	---	---
Diabetes^d^						
Y	33	10.9	22	11.3	11	10.0
N	271	89.1	172	88.7	99	90.0
HIV						
Y	8	2.6	8	4.1	---	---
N	186	61.2	186	95.6	---	---

^a^Missing values included: rifampin resistance (*n* = 111); HIV (*n* = 110). Reported percentages include missing values in the denominators.

^b^TB status determine by Xpert MTB/RIF

^c^Median 40; min 18; max 60; IQR 28–47

^d^According to patient self-report

Among patients with confirmed TB (by Xpert; *n* = 194), 11.3 % had diabetes and 10.8 % were rifampin resistant (Table 1). 27.3 % of patients with diabetes had rifampin resistance, compared to 8.8 % of patients with no diabetes (*p* < 0.01). Rifampin resistance was associated with diabetes (adjusted risk ratio [aRR] 2.96 [95 % confidence intervals {CI}: 1.30-6.74]), previous TB treatment (aRR 3.33 [95 % CI 1.53–7.22]), and positive AFB (aRR 3.14 [95 % CI: 1.03–9.58]) (Table 2).

**Table 2:** Factors associated with rifampin-resistant *Mycobacterium tuberculosis* based on Xpert MTB/RIF assay

	Rifampin resistance			
	Univariate		Multivariate	
	RR (95 % CI)	*P*	aRR (95 % CI)	*P*
Age (years)	0.99 (0.96–1.03)	0.78	---	---
Sex (male)	0.58 (0.18–1.88)	0.37	---	---
Diabetes^a^	3.11 (1.35–7.17)	<0.01	2.96 (1.30–6.74)	<0.01
Previous TB Treatment	3.52 (1.58–7.84)	<0.01	3.33 (1.53–7.22)	<0.01
BCG Vaccination	0.60 (0.26–1.36)	0.22	---	---
TB Symptoms (weeks)^b^	1.00 (0.97–1.03)	0.81	---	---
QFT-G	2.02 (0.50–8.22)	0.33	---	---
AFB	3.60 (0.87–14.91)	0.08	3.14 (1.03–9.58)	0.05

Abbreviations: *AFB* Acid-fast bacilli sputum smear microscopy, *BCG* Bacille Calmette-Guérin, *CI* confidence interval, *TB* tuberculosis, *QFT-G* Quantiferon-TB Gold In-Tube, *RR* risk ratio, *aRR* adjusted risk ratio

^a^According to patient self-report

^b^Time (weeks) since initial onset of symptoms suggestive of TB, including persistent cough, fever, weight loss

Considering Xpert as the gold standard, AFB had 72.2 % (95 % CI: 65.3–78.4) sensitivity and 96.4 % (95 % CI: 91.0–99.0) specificity (Additional file 2: Table S1). In contrast, QFT-G had higher sensitivity (82.5 % [95 % CI: 76.8–88.2], but lower specificity (37.0 % [95 % CI: 27.1–46.8]; Additional file 2: Table S1). With Xpert as the reference, AFB had higher positive predictive value (PPV; 97.2 % [95 % CI: 93.0–99.2]) and negative predictive value (NPV; 66.3 % [95 % CI: 58.4–73.5]) than QFT-G (PPV 70.9 % [95 % CI: 64.5–77.2], NPV 53.1 % [95 % CI: 40.9–65.4]; Additional file 2: Table S1). Similarly, AFB (relative to Xpert) had a higher AUC (0.84; Additional file 3: Figure S1A) compared to QFT-G (0.60; Additional file 3: Figure S1B).

## Discussion

We observed an association between rifampin resistance and diabetes among adult patients with active TB in an urban setting of India. This finding corroborates previous studies that showed greater risk of MDR-TB among patients with TB and diabetes, compared to those without diabetes [8]. Moreover, other studies confirmed associations between Type 2 diabetes and MDR-TB [9] as well as delayed time to sputum smear conversion [10]. Another study among patients with TB found approximately 50 % reductions of rifampin and isoniazid plasma concentrations (both *p* < 0.05), among patients with diabetes, compared to patients without diabetes [11]. However in contrast, other studies showed no associations between TB drug resistance and diabetes, or differences in treatment outcomes based on TB drug resistance and diabetes status [12].

Rifampin resistance was observed in 10.8 % of patients with TB in this study. Directly comparing this estimate to other estimates is challenging given the limited recent nationally-representative data from India [13]. For example, among patients with suspected TB in Maharashtra, rifampin resistance ranged between 9 % (rural) to 51 % (urban) [14].

The comparative performances of AFB smear and QFT-G (relative to Xpert) have also been considered by previous literature as a diagnostic test for active TB. A meta-analysis found that for the detection of active TB in blood and extrasanguinous fluids, QFT-G had a pooled sensitivity of 80 % (95 % CI: 75–84 %) for culture-confirmed cases [15], which is similar to the 82.5 % sensitivity in our study. However the observed specificity was much lower (37 %). ROC curves also reflect that QFT-G should not be used in diagnosing TB in this setting and AFB smear is less than ideal. Our findings therefore support the continued need for improved diagnostic capacity in order to effectively address TB.

Study limitations included the cross-sectional data, which does not allow for causal inference or the consideration of time (as well as disease progression). Although we controlled for certain covariates, residual confounding remains possible. This study was a secondary analyses of data collected for a parent study. Consequently the limitations included the unavailability of TB culture data and any biological indicator of diabetes (glycosylated hemoglobin, fasting glucose). Although diabetes was based on self-report and not confirmed by another biological indicator, this would likely result in non-differential misclassification. Additionally, these data were insufficient to distinguish between Type 1 versus Type 2 diabetes or account for appropriate treatment. However in contrast, a validity study showed that self-report of diabetes is fairly consistent among patients receiving treatment [16].

Strengths of this study include that TB and rifampin resistance were detected by Xpert, which has high sensitivity (>90 %) and specificity (>95 %) for rifampin detection [17], and was endorsed by the WHO in 2010. Rifampin resistance has been considered a potential indicator for MDR-TB [18], given that a large proportion of TB strains with rifampin resistance are also isoniazid resistant. Furthermore, rifampin is a key antituberculous drug in standard TB treatment.

## Conclusions

In rural Andhra Pradesh, self-reported diabetes was associated with 3.0-fold higher risk of rifampin resistance among patients with confirmed TB. AFB had lower sensitivity (72.2 vs. 82.5 %) and higher specificity (96.4 vs. 37.0 %) compared to QFT-G for diagnosing TB.

Our findings on the association between diabetes and rifampin resistance highlight the need for integrated diabetes surveillance in TB programs, particularly in similar settings going through the epidemiological transition. Furthermore, quantifying the disparities in results of different TB diagnostic methods is critical for establishing a global research environment in which results from different studies can be accurately compared to draw conclusions.

## Additional files

Additional file 1:
**Strobe Checklist.** (DOCX 58 kb) Additional file 2: Table S1. Sensitivity, specificity, and predictive values of acid-fast bacilli smear microscopy and QuantiFERON-TB Gold compared with Xpert MTB/RIF. (DOCX 99 kb) Additional file 3:
**Figures S1 A & B.** Receiver operator characteristic curves for AFB and QFT-G compared to Xpert MTB/RIF. (DOC 101 kb)

## Abbreviations

AFB: Acid-fast bacilli sputum smear microscopy
aRR: Adjusted risk ratio
AUC: Area under the curve
BCG: Bacille Calmette-Guérin
CI: Confidence interval
HIV: Human immunodeficiency virus
MDR-TB: Multidrug-resistant TB
MTB: Mycobacterium tuberculosis
QFT-G: QuantiFERON-TB Gold In-Tube
ROC: Receiver operator characteristic
RR: Risk ratio
SD: Standard deviation
TB: Tuberculosis
Xpert: Xpert MTB/RIF

## References

[1] Dooley KE, Chaisson RE (2009). Tuberculosis and diabetes mellitus: convergence of two epidemics. Lancet Infect Dis.

[2] Baker MA, Harries AD, Jeon CY, Hart JE, Kapur A, Lönnroth K, Ottmani S-E, Goonesekera SD, Murray MB (2011). The impact of diabetes on tuberculosis treatment outcomes: a systematic review. BMC Med.

[3] Jeon CY, Murray MB (2008). Diabetes mellitus increases the risk of active tuberculosis: a systematic review of 13 observational studies. PLoS Med.

[4] World Health Organization (2014). Global Tuberculosis Report 2014.

[5] Nijland HM, Ruslami R, Stalenhoef JE, Nelwan EJ, Alisjahbana B, Nelwan RH, van der Ven AJ, Danusantoso H, Aarnoutse RE, van Crevel R (2006). Exposure to rifampicin is strongly reduced in patients with tuberculosis and type 2 diabetes. Clin Infect Dis.

[6] Weiner M, Benator D, Burman W, Peloquin CA, Khan A, Vernon A, Jones B, Silva-Trigo C, Zhao Z, Hodge T (2005). Association between acquired rifamycin resistance and the pharmacokinetics of rifabutin and isoniazid among patients with HIV and tuberculosis. Clin Infect Dis.

[7] Omran AR (2005). The epidemiologic transition: a theory of the epidemiology of population change. Milbank Q.

[8] Bashar M, Alcabes P, Rom WN, Condos R (2001). Increased incidence of multidrug-resistant tuberculosis in diabetic patients on the Bellevue Chest Service, 1987 to 1997. Chest J.

[9] Perez-Navarro LM, Fuentes-Dominguez FJ, Zenteno-Cuevas R (2014). Type 2 diabetes mellitus and its influence in the development of multidrug resistance tuberculosis in patients from southeastern Mexico. J Diabetes Complications.

[10] Chang JT, Dou HY, Yen CL, Wu YH, Huang RM, Lin HJ, Su IJ, Shieh CC (2011). Effect of type 2 diabetes mellitus on the clinical severity and treatment outcome in patients with pulmonary tuberculosis: a potential role in the emergence of multidrug-resistance. J Formos Med Assoc.

[11] Babalik A, Ulus IH, Bakirci N, Kuyucu T, Arpag H, Dagyildizi L, Capaner E (2013). Plasma concentrations of isoniazid and rifampin are decreased in adult pulmonary tuberculosis patients with diabetes mellitus. Antimicrob Agents Chemother.

[12] Magee MJ, Kempker RR, Kipiani M, Tukvadze N, Howards PP, Narayan KM, Blumberg HM (2014). Diabetes mellitus, smoking status, and rate of sputum culture conversion in patients with multidrug-resistant tuberculosis: a cohort study from the country of Georgia. PLoS One.

[13] Zignol M, Gemert WV, Falzon D, Sismanidis C, Glaziou P, Floyd K, Raviglione M (2012). Surveillance of anti-tuberculosis drug resistance in the world: an updated analysis, 2007–2010. Bull World Health Organ.

[14] Almeida D, Rodrigues C, Udwadia ZF, Lalvani A, Gothi GD, Mehta P, Mehta A (2003). Incidence of multidrug-resistant tuberculosis in urban and rural India and implications for prevention. Clin Infect Dis.

[15] Sester M, Sotgiu G, Lange C, Giehl C, Girardi E, Migliori GB, Bossink A, Dheda K, Diel R, Dominguez J (2011). Interferon-gamma release assays for the diagnosis of active tuberculosis: a systematic review and meta-analysis. Eur Respir J.

[16] Margolis KL, Lihong Q, Brzyski R, Bonds DE, Howard BV, Kempainen S, Simin L, Robinson JG, Safford MM, Tinker LT (2008). Validity of diabetes self-reports in the Women’s Health Initiative: comparison with medication inventories and fasting glucose measurements. Clin Trials.

[17] Boehme CC, Nicol MP, Nabeta P, Michael JS, Gotuzzo E, Tahirli R, Gler MT, Blakemore R, Worodria W, Gray C (2011). Feasibility, diagnostic accuracy, and effectiveness of decentralised use of the Xpert MTB/RIF test for diagnosis of tuberculosis and multidrug resistance: a multicentre implementation study. Lancet.

[18] Lawn SD, Brooks SV, Kranzer K, Nicol MP, Whitelaw A, Vogt M, Bekker L-G, Wood R (2011). Screening for HIV-associated tuberculosis and rifampicin resistance before antiretroviral therapy using the Xpert MTB/RIF assay: a prospective study. PLoS Med.

