# Supplementary material for: Rifampin resistance and diabetes mellitus in a cross-sectional study of adult patients in rural South India
Source: BMC Infect Dis. 2015 Oct 26;15:451. doi: 10.1186/s12879-015-1204-5 (PMC4620627; doi:10.1186/s12879-015-1204-5)
Supplement: Additional file 1: — Strobe Checklist. (DOCX 58 kb) [file 12879_2015_1204_MOESM1_ESM.docx]

Supplementary Table 1 ^a^: Sensitivity, specificity, and predictive values of acid-fast bacilli smear microscopy and QuantiFERON-TB Gold compared with Xpert MTB/RIF

| *% (95 % CI) ^b^* | Xpert MTB/RIF | | | |
| --- | --- | --- | --- | --- |
|  | Sensitivity | Specificity | PPV | NPV |
| AFB | 72.2 (65.3-78.4) | 96.4 (91.0-99.0) | 97.2 (93.0-99.2) | 66.3 (58.4-73.5) |
| QFT-G | 82.5 (76.8-88.2) | 37.0 (27.1-46.8) | 70.9 (64.5-77.2) | 53.1 (40.9-65.4) |
| ^a^ Abbreviations include: acid-fast bacilli sputum smear microscopy (AFB); Quantiferon-TB Gold (QFT-G)  ^b^ 95% confidence intervals are Wald (AFB) or exact (QFT-G) | | | | |
